# The interaction between wheat roots and soil pores in structured field soil

**DOI:** 10.1093/jxb/eraa475

**Published:** 2020-10-16

**Authors:** Hu Zhou, William R Whalley, Malcolm J Hawkesford, Rhys W Ashton, Brian Atkinson, Jonathan A Atkinson, Craig J Sturrock, Malcolm J Bennett, Sacha J Mooney

**Affiliations:** 1 School of Biosciences, University of Nottingham, Loughborough, Leicestershire, UK; 2 State Key Laboratory of Soil and Sustainable Agriculture, Institute of Soil Sciences, Chinese Academy of Sciences, Nanjing, PR China; 3 Rothamsted Research, Harpenden, UK; 4 University of Antwerp, Belgium

**Keywords:** Biopore, genotype, macropore, subsoil, wheat, X-ray computed tomography

## Abstract

Wheat (*Triticum aestivum* L.) root growth in the subsoil is usually constrained by soil strength, although roots can use macropores to elongate to deeper layers. The quantitative relationship between the elongation of wheat roots and the soil pore system, however, is still to be determined. We studied the depth distribution of roots of six wheat varieties and explored their relationship with soil macroporosity from samples with the field structure preserved. Undisturbed soil cores (to a depth of 100 cm) were collected from the field and then non-destructively imaged using X-ray computed tomography (at a spatial resolution of 90 µm) to quantify soil macropore structure and root number density (the number of roots cm^–2^ within a horizontal cross-section of a soil core). Soil macroporosity changed significantly with depth but not between the different wheat lines. There was no significant difference in root number density between wheat varieties. In the subsoil, wheat roots used macropores, especially biopores (i.e. former root or earthworm channels) to grow into deeper layers. Soil macroporosity explained 59% of the variance in root number density. Our data suggested that the development of the wheat root system in the field was more affected by the soil macropore system than by genotype. On this basis, management practices which enhance the porosity of the subsoil may therefore be an effective strategy to improve deep rooting of wheat.

## Teaser

The development of the wheat root system in the field was more affected by soil macropore arrangement than by genotype.

## Introduction

Drought stress is a major limitation to wheat (*Triticum aestivum* L.) production globally (Fahad *et al.*, 2017; Mäkinen *et al.*, 2018; Leng and Hall, 2019). Any approach that will lead to a deep root system, allowing access to water stored in the subsoil, is a promising strategy to adapt to a water-limited environment (Gao *et al.*, 2016*a*; Morris *et al.*, 2017; Friedli *et al.*, 2019). Even in the UK, limitations in water availability can reduce wheat yield (Dodd *et al.*, 2011). White *et al.* (2015) measured the root length density of 17 commercial winter wheats in the UK and suggested that the poor rooting of modern varieties might be responsible for the yield stagnation experienced in the UK since the 1990s, due to poor access to water. Manschadi *et al.* (2006) found that 1 mm of additional water extracted by deep roots during grain filling could increase grain yield by 55 kg ha^–1^. An understanding of root traits and how these traits interact with their environment is needed to breed wheat with deep-rooting traits that can achieve high productivity under drought stress (Comas *et al.*, 2013).

The deep-rooting traits of wheat have been a subject of great interest, especially in recent years, to aid wheat breeding (Jung and McCouch, 2013; Friedli *et al.*, 2019). Aziz *et al.* (2017) found that modern wheat varieties had a reduced root length by comparing wheat varieties released between 1958 and 2007 in Australia using data from repacked rhizotrons. Friedli *et al.* (2019) studied 14 bread wheat genotypes released in the last 100 years in Switzerland, grown in repacked soil, and found that rooting depth was correlated with plant height in well-watered conditions. Under drought stress, the relationship between plant height and root depth was less clear, which was attributed to deeper root growth stimulated by limited water availability.

While root growth studies based on repacked soil have provided important data on plant architecture, it is critical to further understand the effect of soil structure on root growth under field conditions. In the field, soil strength increases with depth due to the effects of overburden pressure (i.e. pressure due to the weight of soil) (Gao *et al.*, 2016*b*); this is a ubiquitous phenomenon and it means that soil strength will increase with depth even if there is no soil compaction (Gao *et al.*, 2016*b*), as illustrated in Supplementary Fig. S1 at *JXB* online. Valentine *et al.* (2012) showed that elongation of roots in packed soil is much greater than in field soil, providing evidence that field and repacked soils provide very different environments for root growth. White and Kirkegaard (2010) reported that deep wheat roots were found in pores, implying that soil structure, or the existence of a continuous pore network at depth, might be as important as genotype in determining rooting depth. Recently, we have shown in a laboratory study that, in strong soil, wheat roots will preferentially elongate in macropores (Atkinson *et al.*, 2020). New research to establish and quantify relationships between macropores and root elongation in the field is urgently needed.

The quantitative assessment of the three-dimensional (3D) soil macropore network has become tractable due to the use of X-ray computed tomography (CT) (Mooney 2002; Luo *et al.*, 2008; Naveed *et al.*, 2013). The technique is also able to provide a means for the quantification of 3D root systems embedded within the soil (Mairhofer *et al.*, 2017; Atkinson *et al.*, 2019). The objectives of this study were to (i) investigate the deep-rooting traits of six wheat genotypes; (ii) visualize and measure the macropore characteristics of soil cores from the field to a depth of 100 cm; and (iii) quantify the relationship between the wheat root system and soil macroporosity. For convenience and practicality, the vast majority of previous studies have been based on repacked soil columns, rhizotrons, or model systems (e.g. sand culture, vermiculite, hydroponics, etc.), and to date field data are more limited. We sought to understand how the natural macropore structure in arable soils affected the root growth and root elongation of wheat to a depth of 100 cm.

## Materials and methods

### Experimental site and experimental design

The experiments were conducted on Broadmead field at Woburn experimental farm, Bedfordshire, UK (52°01'11.2''N, 0°35'30.4''W). In this field, soil in the 0–40 cm layer was a Fluvisol with a silt–clay loam texture. There was a vertical gradient in texture to a depth of 100 cm, with deeper layers having a greater sand content (Hodgkinson *et al.*, 2017). The surface layer (~30 cm) had a higher organic matter content. To a depth of 60 cm, the bulk density of the soil did not change greatly, and was ~1.2 g cm^–3^. Soil properties are summarized in Table 1. The soil profile on Broadmead is consistent with the description of a soil profile by Weir *et al.* (1984) that would be expected to produce high yields of winter wheat.

**Table 1.** Description of the topsoil (0–40 cm below the surface) properties of Woburn experimental field station, Bedfordshire, UK

Property	Units	
Location	Latitude	52°01'06''N
	Longitude	00°35'30''W
Soil type	SSEW group^*a*^	Typical alluvial Gley soil
	SSEW series^*b*^	Eversley
	FAO	Fluvisol
Sand (2000–65 μm)	g g^–1^ dry soil	0.538
Silt (63–2 μm)	g g^–1^ dry soil	0.203
Clay (<2 μm)	g g^–1^ dry soil	0.260
Texture	SSEW class	Sandy clay loam
Particle density	g cm^–3^	2.587
Organic matter	g g^–1^ dry soil	0.038

SSEW, Soil Survey of England and Wales.

^*a*^ Avery (1980).

^*b*^ Clayden and Hollis (1984).

The field experiment had 504 separate 9 m×1.8 m plots, divided into three fully randomized blocks, with each block containing 168 plots of different wheat lines and one fallow plot. The six wheat lines of interest in this study were randomly arranged within each block. The plots were sown on 10 October 2017. The field site was rain fed with no additional irrigation. Husbandry of the crops followed standard agronomic protocols for the UK, with inputs to ensure adequate nutrition, weed, pest, and disease control.

The six genotypes sampled were near isogenic line (NIL) Rht-B1a (tall), NIL Rht-B1c (dwarf), Cadenza, Paragon, Xi19, and Shamrock. Previously we have found that when differences in rooting depth are found, NIL Rht-B1c is amongst the wheats with the deepest roots (Hodgkinson *et al.*, 2017; Bai *et al*, 2019). Cadenza and Paragon have both been used as reference wheats for comparison of traits. Shamrock was selected for study because rhizotron studies have identified this wheat to be deep rooting (Clarke *et al.*, 2017). Xi19 is a Cadenza×Rialto cross and semi-dwarf, with the potential for high yields.

### Sampling

**Fig. 1.**
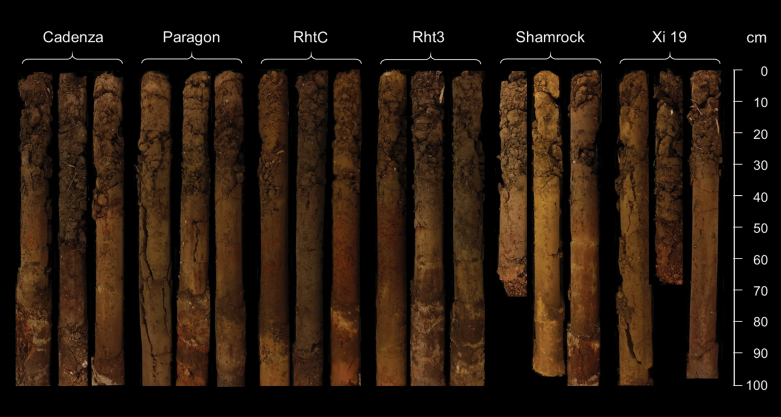
Illustration of the 18 soil cores (six wheat genotypes, three replicates, 9 cm in diameter) taken from the field experiment.

Cylindrical soil cores were collected on 26 June 2018 using a soil column cylinder auger (VanWalt Ltd, Surrey, UK). The cores were ~100 cm long and 9 cm in diameter. One core was taken from ~100 cm in from the end of each wheat plot and a total of 18 cores were collected (Fig. 1). Once extracted, cores were placed in a 105 cm length of polyethylene guttering, wrapped in sealed polyethylene bags, transported to the University of Nottingham, and stored at 4 °C before X-ray CT scanning.

### X-ray CT scanning and image analysis

Soil cores were stabilized, by placing inside a 20 cm Ø plastic tube ~80 cm in height, in a vertical orientation using foam packing material. These tubes were scanned using a Phoenix v|tome|x L Custom® μCT scanner (GE Sensing and Inspection Technologies, Wunstorf, Germany) at the Hounsfield facility, University of Nottingham, UK. The voltage and current used were 290 kV and 2700 µA, respectively. A spatial resolution of 90 μm was used in all scans. During the scan, the specimen stage rotated through 360° at a rotation step increment of 0.129°, collecting a total of 2800 projection images. To reduce image noise, each projection image was an average of five frames, each acquired with an exposure time of 200 ms. To avoid oversaturation of the detector panel, a 0.5 mm copper filter was used over the exit window of the X-ray tube and the detector panel. Due to the height of the field core (100 cm), eight separate scans were required to image the entire core, with an overlap of ~10 mm between each adjacent scan, resulting in a total scan time of 7 h per core. The eight scans per core were reconstructed using the ‘multi-scan’ reconstruction feature in Phoenix datos x software (GE Sensing and Inspection Technologies) to give a 3D 16-bit greyscale volume. Each XY slice in the volume was 2000×2000 voxels in size; however, the length (Z) varied between samples due to slight differences in the length of each core. The 3D volume files were exported as 16-bit greyscale slices (tiff format) using VG StudioMAX 3.0 software. A fully scanned core, with a height of 100 cm, had ~11 050 individual image slices, with a total data size of ~82 gigabytes (GB).

**Fig. 2.**
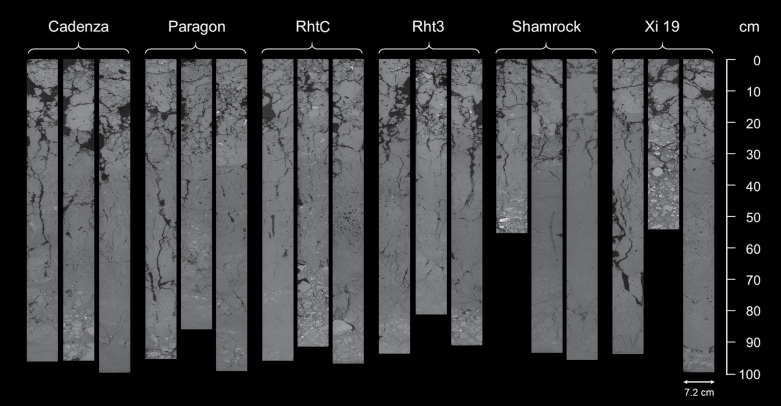
Soil longitudinal sections taken from the centre of the soil cores. Darker colours are soil pores, brighter grey colours are soil matrix.

**Fig. 3.**
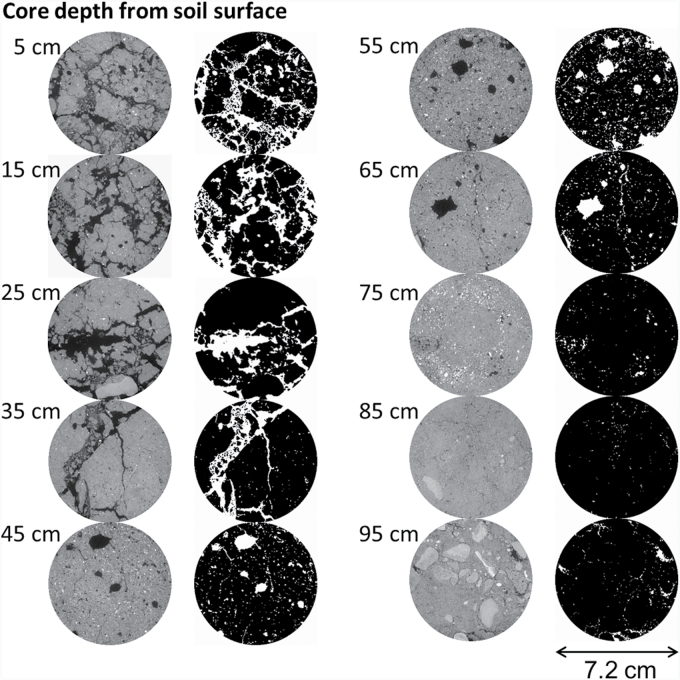
Examples of the horizontal greyscale image slices (left column) and the corresponding segmented binary slices (right column) of a soil core (sample #140) at 5 cm incremental depths from the soil surface.

A region of interest (ROI) was selected from the central part of each core to discount any potential disturbance at the edge of the samples that might have occurred during sampling. The diameter of the ROI was 800 voxels, while the length of the ROI varied depending on the length of the samples. The longitudinal sections of the core images are shown in Fig. 2. One pass of a median filter (3×3) was used to remove noise. The size of datasets was considerably greater than in any comparable work. Unfortunately, using the sophisticated algorithms currently available for the segmentation of soil structure data such as indicator kriging via the 3DMA-Rock software (Oh and Lindquist, 1999) was not possible due to the size of the image data. We determined that we could obtain a comparable result using a user-defined global threshold value. To minimize bias, all the segmentations were conducted by the same operator. Examples of the segmented binary slices from a core sample and the corresponding greyscale slices for different depth intervals are shown in Fig. 3.

Soil macroporosity of each slice was calculated by dividing the pore area (white phase as shown in the binary images in Fig. 3) by the total area of the ROI. All the segmented pores from CT images were larger than the minimum resolution of the images (90 μm). Therefore, they are regarded as macropores in this study and thus the term ‘macroporosity’ has been used. The vertical distribution of soil macroporosity was assessed at 10 cm depth intervals to correspond to the root data as discussed below. The average macroporosity of each 10 cm soil section from the surface was calculated from the average of the macroporosity of corresponding slices. In this study, the calculated macroporosity included pores which were ‘root filled’.

### Root counting

**Fig. 4.**
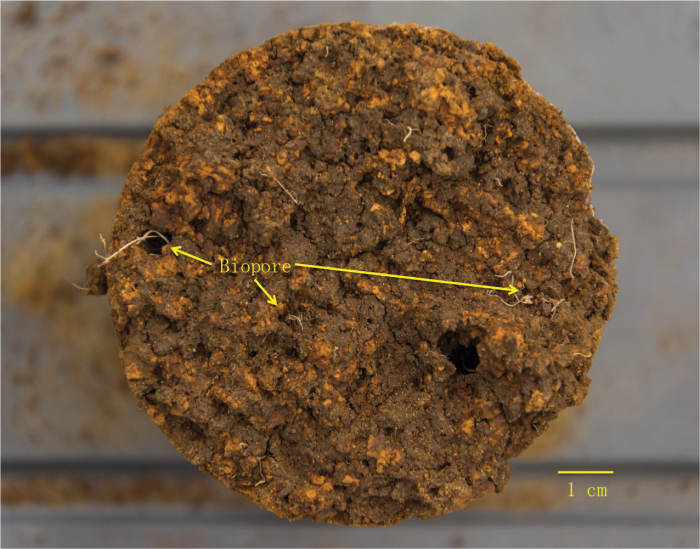
A horizontal soil surface at 65 cm depth of a soil core (sample #273) cleaned for root counting.

Due to a combination of the (i) spatial resolution (a function of the large core sizes used in this study), (ii) contrast resolution (i.e. the ability to discern objects of low density from their background), (iii) the signal to noise ratio of the images, (iv) the size of the wheat roots (with some as thin as 100 µm), and (v) the way in which they were frequently embedded within the heterogenous soil matrix, standard approaches of root segmentation were unsuccessful. Tools such as RooTrak (Mairhofer *et al*., 2012), Root1 (Flavel *et al.*, 2017), and Rootine (Gao *et al.*, 2019) which have been previously demonstrated to be effective in smaller sized columns and in repacked homogenous soil were evaluated but unable to adequately segment the roots, as was a manual root segmentation approach; thus we had to use a manual root counting method. Root counting procedures were modified from White and Kirkegaard (2010) and (Hodgkinson *et al.* (2017). Soil cores were sequentially broken transversely (horizontal to the original soil surface) from the top to different depths at an interval of 10 cm. The selected depths were 5, 15, 25, 35, 45, 55, 65, 75, 85, and 95 cm. During core breaking, great care was taken to sample the selected depth with the estimated variations between the actual and selected depth <1 cm. At each of the selected depths, the soil surface was cleaned and exposed, as shown in Fig. 4. Root numbers were counted manually on the selected surfaces. All observed fresh roots were recorded. A root was noted as growing inside a pore if it was surrounded by pore space that was twice as large as the root size. The number of roots growing inside a pore was only counted for the subsoil (deeper than 35 cm). Root number density was calculated as the number of roots cm^–2^. The maximum rooting depth was determined as the deepest layer with roots observed.

### Statistical analysis

Statistical analysis was performed using RStudio software (R version 3.3.3). The normality of residuals and assumptions of the homogeneity of variances were checked by the Shapiro–Wilk and Bartlett tests, respectively, prior to any further statistical tests. Two-way ANOVA was conducted with genotype and soil depth as the two factors, and their interactions were also examined. Post-hoc analysis was performed by the Tukey HSD test for significant differences between treatments at *P*<0.05. Correlation between root number and macroporosity was determined using Pearson’s correlation coefficients.

## Results

### Soil macropore system is influenced by depth not wheat genotype

**Fig. 5.**
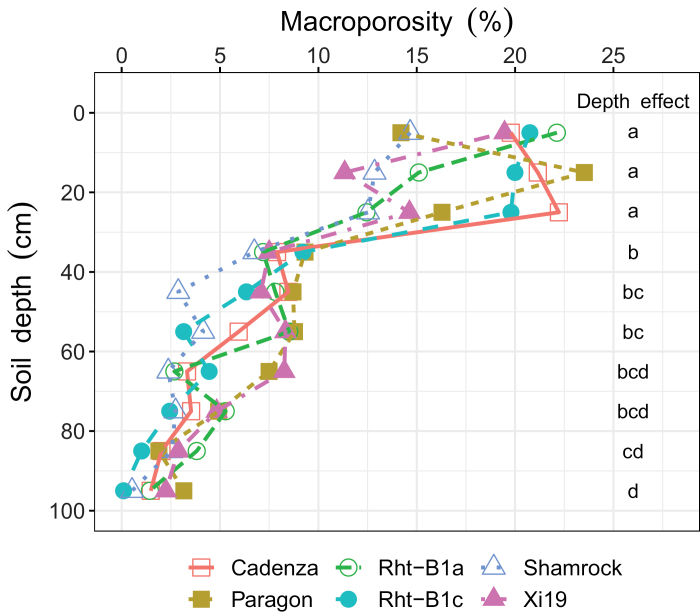
Soil macroporosity profiles of different wheat genotype treatments to a depth of 100 cm. Different letters below ‘Depth effect’ indicate significant difference of soil macroporosity between the corresponding soil depth (*P*<0.05).

Soil macroporosity was not significantly different between the wheat genotypes but it decreased significantly with soil depth (*P*<0.05). There was no significant effect of the interaction between genotype and soil depth on macroporosity (*P*>0.05). The vertical distribution of soil macropores can be observed in Fig. 2, and detailed horizontal illustrations of each depth for a representative example soil core are presented in Fig. 3. Visual observation revealed that macropores in the plough layer (0–30 cm, as shown in Figs 2 and 3) were typically a combination of cracks, biologically originated pores (i.e. biopores with tubular shapes and continuous structure), and packing pores (pores resulting from the packing of soil particles or aggregates). For the subsoil, the macropores were mainly biopores, as indicated in Fig. 4, formed by decayed roots or earthworm channels. Soil macroporosity in the plough layer was significantly higher than in the subsoil (*P*<0.05, Fig. 5). A sharp decrease in macroporosity was observed from 16.3% at the 25 cm depth to 8.0% at the 35 cm depth for all the cores. A further, but gradual, decrease was observed in the deep layers, with the lowest macroporosity observed in the deepest layer (1.5%).

### Vertical distribution of root number densities did not differ between wheat genotypes

**Fig. 6.**
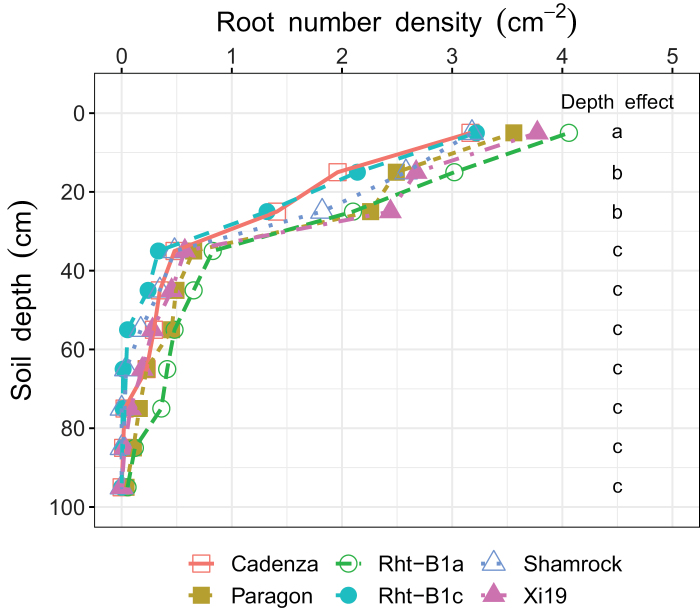
Vertical distribution of root number density of different wheat genotypes to a depth of 100 cm. Root number density: number of roots cm^–2^. Different letters below ‘Depth effect’ indicate significant difference of root number density between the corresponding soil depth (*P*<0.05).

Root number density was not significantly different between wheat genotypes nor were there any genotype×depth interactions (*P*>0.05). The effect of depth on root number density was significant (*P*<0.05). The number of roots was highest in the top layer (0–10 cm, 3.5 roots cm^–2^), followed by the 10–20 cm and 20–30 cm depth (1.9–2.5 roots cm^–2^), and lowest in the subsoil (>30 cm depth, 0–0.6 roots cm^–2^) (Fig. 6).

The maximum wheat rooting depths for the different wheat varieties ranged from 45 cm to 95 cm, with an average of 72.2 cm (Supplementary Fig. S2). There was no significant difference in the maximum rooting depth for the six wheat genotypes.

### Interaction between wheat root growth and soil macropore

**Fig. 7.**
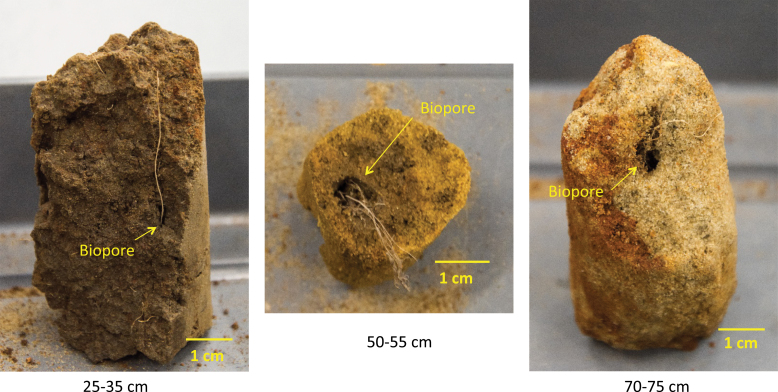
Examples of wheat roots grown in macropores at different depths (>30 cm).

**Fig. 8.**
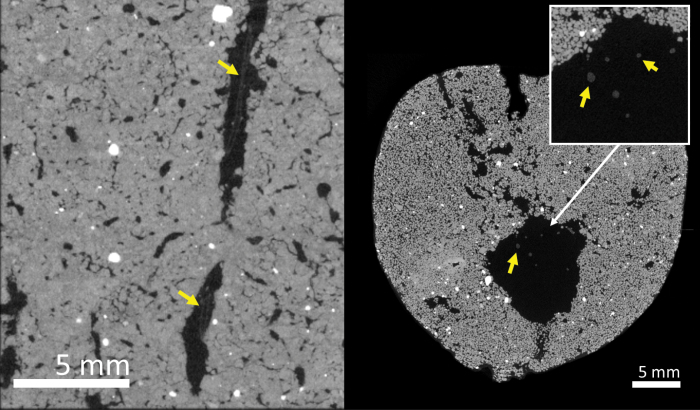
CT images showing wheat rooting inside macropores at 35–45 cm depth in vertical orientation (left) and at 50–60 cm depth in cross-section (right) at a resolution of 20 µm. Yellow arrows denote wheat roots inside soil pores.

**Fig. 9.**
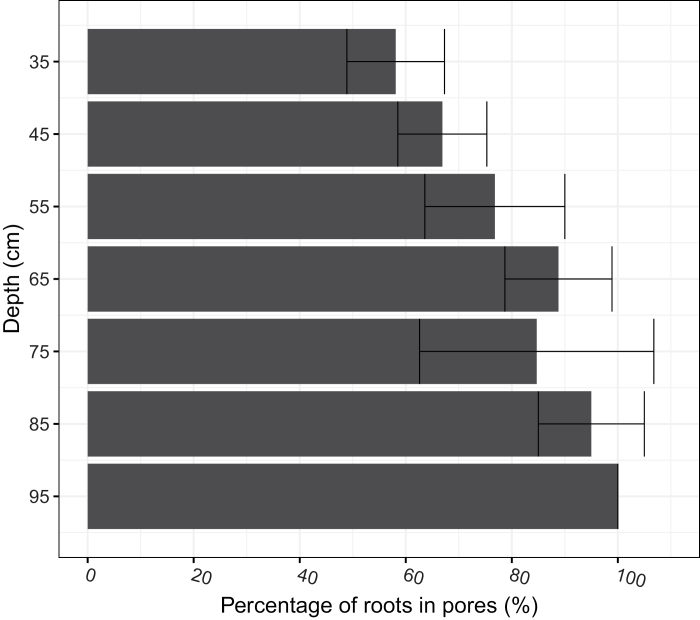
Vertical distribution of the percentage of roots in pores from 35 cm to 95 cm. Percentage of roots in pores is the percentage of the number of roots found in pores relative to the total number of roots for the specific depth. Data of the six wheat genotypes showed no significant difference and their averages were therefore presented. Error bars indicate the SD.

**Fig. 10.**
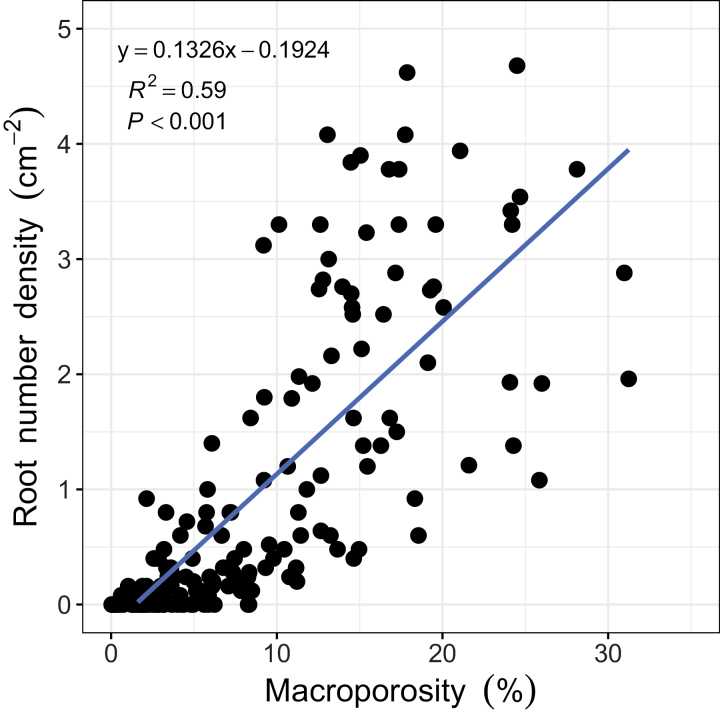
Relationship between wheat root number density and macroporosity at the depth of 0–100 cm. Root number density: number of roots cm^–2^.

Roots were found growing in macropores in the subsoil of all the soil cores (Fig. 7). Cross-section images revealed that roots in the subsoil tended to follow the path of macropores to penetrate these soil layers (Fig. 8). At a depth >35 cm, the proportion of roots confined in the pre-existing macropores ranged between 50% and 100%, with an average of 81.5%, showing no significant difference between wheat genotypes (Fig. 9). The number of roots was positively correlated with macroporosity, and a linear regression model explained 59% (*P*<0.001) of the variation of root number density (Fig. 10).

## Discussion

### Rooting mechanisms

The deep rooting characteristics of wheat are important traits that impact nutrient and water acquisition from the subsoil (Atkinson *et al.*, 2019; Bai *et al.*, 2019; Guo *et al.*, 2020). However, Guo *et al.* (2020) report that low heritability in deep rooting was caused by very large environmental variance due to spatial effects and measurement errors. It is therefore important to understand how environmental factors contribute to variability in root depth. Wheat root elongation in the subsoil is commonly limited by soil strength (Gao *et al.*, 2016*a*) or by high levels of water saturation (Hodgkinson *et al.*, 2017). Pre-existing macropores can provide paths for roots to reach deeper layers and bypass strong soil (Fahad *et al.*, 2017). We found in the subsoil that most of the roots grew in the macropores (Fig. 9), to the extent that macroporosity explained 59% of the variance in root number density (Fig. 10). This finding is consistent with previous studies (White and Kirkegaard 2010; Hodgkinson *et al.*, 2017) based on direct observation of the surface of fractured soil cores. The macropores that deep roots utilize were mostly biopores formed from root decomposition or faunal burrowing such as that of earthworms, which can be visually determined from their 3D morphology (Figs 7, 8). The biopores not only provided paths with lower mechanical resistance, but also altered the physical, chemical, and biological properties of the surrounding soil that benefit root growth (Kautz *et al.*, 2013; Banfield *et al.*, 2017). In previous studies, artificial pores have been created to study root behaviour in soil (Pfeifer *et al.*, 2014; Colombi *et al.*, 2017; Atkinson *et al.*, 2020). In these examples, the soil was artificially packed; the soil around artificial pores is most likely to have similar chemical properties to the bulk soil. An exception is the work of Stirzaker *et al.* (1996), who found that roots were more likely to occupy old root channels compared with artifical pores. In the field, the soil around biopores is likely to have distinct physico-chemical properties relative to the bulk soil (Kautz *et al.*, 2013; Haas and Horn, 2018). The biopores in the subsoil are less likely to be disturbed by tillage and, because they tend to have a vertical orientation, they are less sensitive to the effects of compaction and can remain intact for many years.

Although the growth of roots in macropores has been previously reported, our understanding of the quantitative relationship between macropores and roots is still limited. Quantification of the 3D soil macropore system has been widely used in recent years with the rapid development of X-ray CT imaging (Luo *et al.*, 2008; Naveed *et al.*, 2013; Rab *et al.*, 2014; Zhang *et al.*, 2018). However, most studies have only focused on a limited depth of soil, and usually no deeper than 40 cm (Luo *et al.*, 2008; Zhang *et al.*, 2018). Here we reveal the undisturbed macropore system of an arable soil to a depth of 100 cm for the first time. We show that soil macroporosity changes with soil depth but does not differ between wheat genotypes. Macroporosity was typically high in the upper 0–30 cm (usually the zone of cultivation) but decreased sharply in the subsoil (>30 cm). A positive correlation was found between root number density and soil macroporosity, indicating that macroporosity had a significant impact on the wheat rooting. The results are consistent with previous laboratory simulation experiments (Colombi *et al.*, 2017), as well as observation of field samples (White and Kirkegaard 2010), which suggest that the generation of macropores in the subsoil is an effective way to enhance wheat rooting. This is important information for future plant breeding programmes, and also soil management strategies, such as those associated with sustainable intensification and conservation agriculture (Williams and Weil, 2004).

We found that soil macroporosity did not vary between the different wheat lines. However, previous studies have shown that plant roots can modify the soil pore system via different mechanisms, including mechanically translocating soil particles/aggregates, increasing soil aggregation through root exudate release and related biologically activities, and root decomposition (Hallett *et al.*, 2009; Bodner *et al.*, 2014). It is likely that this has impacts on soil hydraulic behaviour. Naveed *et al.* (2018) recently showed that plant exudates from barley, maize, and chai can increase soil hardness and decrease soil wetting rates through water repellency. As different root architectures, root sizes, exudate properties, and presence or absence of root hairs can induce different responses in the soil pore system, it is reasonable to postulate that different wheat genotypes might form and deform the soil pore system in contrasting fashions. However, our results did not support this idea at the macropore scale. This could be either because (i) all the wheat lines have a similar effect on soil structure or (ii) they have no effect on soil structure. The latter possibility, at depth, may be due to the impact of overburden pressure in the field which has not been present in almost all previous accounts of root-modifying soil structure in laboratory studies.

Our results support the view that root growth in pre-existing pores is the primary mechanism for deep rooting in wheat. However there remains an urgent need to understand why many pores at depth do not contain roots. It is possible that either they are not continuously connected to the surface or that root architecture in the surface layers does not facilitate pore location by roots. Recently, Fradgley *et al.* (2020) demonstrated significant differences in the near-surface architecture of wheat roots on a selection of modern and historic wheat lines, but whether this translates into differences in deep rooting remains to be determined. It is likely that where biopores are not immediately accessible, roots undertake foraging-like behaviour in order to secure an easier passage through the soil (Atkinson *et al.*, 2020). Roué *et al.* (2020) recently demonstrated, for Arabidopsis, that the root cap size and shape can influence the penetration ability of roots; however, by the resolution adopted in our imaging approach, it was not possible to accurately measure root caps to assess this and therefore is suited to future investigations. Similarly it would be useful to assess the impact of the soil strength at the root–pore interface.

### Rht status and root depth

Wojciechowski *et al.* (2009) showed that the dwarﬁng alleles Rht-B1c, Rht-D1c, and Rht12 had signiﬁcant effects on root length of young seedlings compared with control and semi-dwarf lines. This suggests a role for Rht genes in establishment. In this study, the post-anthesis root number density distribution and rooting depth of six wheat genotypes, which showed contrasting above-ground plant height (Supplementary Fig. S3), exhibited no significant difference between genotypes (Fig. 6). Hodgkinson *et al.* (2017) found that statistical differences in root length distributions were dependent on the season, which was probably related to soil moisture. In a dry year, the NIL Rht-B1c (dwarf) wheat had more roots at depth compared with the NIL Rht-B1a (tall) wheat. More recently, Bai *et al.* (2019) also found that when there were differences between these NILs; Rht-B1c had deeper roots despite the shorter stature. Our results are consistent with rhizotron studies of Friedli *et al.* (2019) who found that deep rooting was not necessarily related to plant height. It is notable that we found that Shamrock, identified by Clarke *et al.* (2017) as a deep rooting wheat, did not apear to have an increased rooting depth. White *et al.* (2015) have suggested that yield stagnation in wheat may be partly explained by a poor rooting of modern wheats compared with older varieties. Given the large environmental impact of soil on rooting depth, deterorating soil conditions arising from contemporary agriculture might well explain poor rooting, as suggested by White *et al.* (2015). It is of concern that Guo *et al.* (2020) observed that in a selction of 84 wheats from high-yielding northern and central European varieties and advanced breeding lines, the heritability of deep rooting was low. This was partly attributed to high environmental variability including heterogeneity in soil type which can often be considerable. As far as we know there is no consistent association between Rht status and rooting depth, except for Rht-B1c, which has a tendency for deep rooting (Hodgkinson *et al.*, 2017; Bai *et al.*, 2019), although it was not observed in this study. Bai *et al.* (2019) speculated that the deep rooting behaviour of Rht-B1c might be related to an increased number of nodal roots associated with a higher tiller number making pore location more efficient, but this remains to be demonstrated.

### Implications for water uptake

Water uptake by wheat roots at depths greater than ~0.5 m is usually very limited (Gregory *et al.*, 1978*a*, *b*; Ober *et al.*, 2014; Zhang *et al.*, 2020). There is a general view that this is at least in part because of a low root density in deeper layers, but Zhang *et al.* (2020) have shown that the hydraulic properties of the rhizosphere can also contribute to low water uptake rates and particularly if any hydrophobicity is induced at the root–soil interface. Root exudates, root decomposition, soil fauna activity, and related microbial processes could increase soil organic carbon and nutrient concentrations of the surrounding soil, which lead to differences in soil mechanical and hydraulic properties associated with modification to soil structure (Whalley *et al.*, 2005; Helliwell *et al.*, 2017; Naveed *et al.*, 2018). Roots can repeatedly use these biopores and therefore their impact on the surrounding soil is likely to be cumulative, forming a unique ‘biopore-sphere’ that has bio-physicochemical properties which differ from those of the bulk soil (Banfield *et al.*, 2017). We observed that individual roots were frequently found in the middle of biopores (Fig. 8), which will result in a poor hydraulic connection between the root and the soil. While clumping of roots within a pore may improve the root–soil contact and increase water uptake from the vicinity of the pore, this does result in a spatial distribution of roots which is less than ideal for water uptake (Tardieu, 1994). The examination of natural biopores from field structured soil is essential for the future study of the effects of biopores on root growth.

### Limitation of the X-ray CT imaging for studying root-soil interactions

X-ray CT has shown considerable promise recently as a tool to study root–soil interactions as it can be used to simultaneously and non-destructively quantify the 3D soil structure (Luo *et al.*, 2008; Naveed *et al.*, 2013; Zhou *et al.*, 2016) and root architecture (Mairhofer *et al.*, 2017; Atkinson *et al.*, 2019). The use of X-ray CT, however, often faces a trade-off between sample size and spatial resolution of the CT images. In this study, the sample size (100 cm long and 9 cm in diameter), greater than most comparable studies, was employed to maximize the observation of wheat roots under field conditions. This limited the image spatial resolution to ~50–90 μm, which made it impossible to automatically extract wheat roots from the CT images. Further research may be able to overcome such limitations via an improved CT machine that can scan larger samples with high spatial resolution and better contrast resolution. However, the increase of spatial resolution raises another issue, namely an increase in data size. In this study, the size of images representing one core is ~80 GB; increasing image resolution from ~90 μm to ~45 μm would be an increase in image data for one core to ~320 GB, which would be very difficult to handle by current image processing and analysis methods. Developing automatic methods to extract root traits from soil cores is therefore urgently needed. The artificial intelligence-based image analysing methods have shown great potential and might be a possible solution (Soltaninejad *et al.*, 2020).

### Conclusions

Undisturbed soil cores, to a depth of 100 cm, from a field experiment were used to investigate and quantify the interaction between soil structure inferred by the macroporosity and wheat rooting patterns for the first time. Soil macroporosity was not significantly affected by wheat genotype but decreased significantly with depth, probably due to soil overburden pressure. The number of roots observed with depth followed a similar trend to the macroporosity. A positive relationship between root number density and soil macroporosity suggests that macropores in the subsoil, most probably biopores, have great impact on rooting behaviour of wheat. Strategies to create biopores such as those which encourage earthworm populations, for example reduced and zero tillage, are likely to be beneficial for improving the utilization of water and nutrients from deeper parts of the soil profile.

## Supplementary data

The following supplementary data are available at *JXB* online.

Fig. S1. A comparison of penetrometer resistance measured in the field with penetrometer resistance measured in the laboratory on cores taken in the field.

Fig. S2. Observed maximum rooting depth of different wheat lines.

Fig. S3. Plant height of different wheat lines.

eraa475_suppl_Supplementary_Figures_S1-S3

## Data Availability

The data supporting the findings of this study are available from the corresponding author (Hu Zhou) upon request.

## Abbreviations

CT: computed tomography
NIL: near isogenic line
3D: three-dimensional
