# Supplementary material for: The interaction between wheat roots and soil pores in structured field soil
Source: J Exp Bot. 2020 Oct 16;72(2):747–56. doi: 10.1093/jxb/eraa475 (PMC7853603; doi:10.1093/jxb/eraa475)
Supplement: eraa475_suppl_Supplementary_Figures_S1-S3 [file eraa475_suppl_supplementary_figures_s1-s3.pdf]

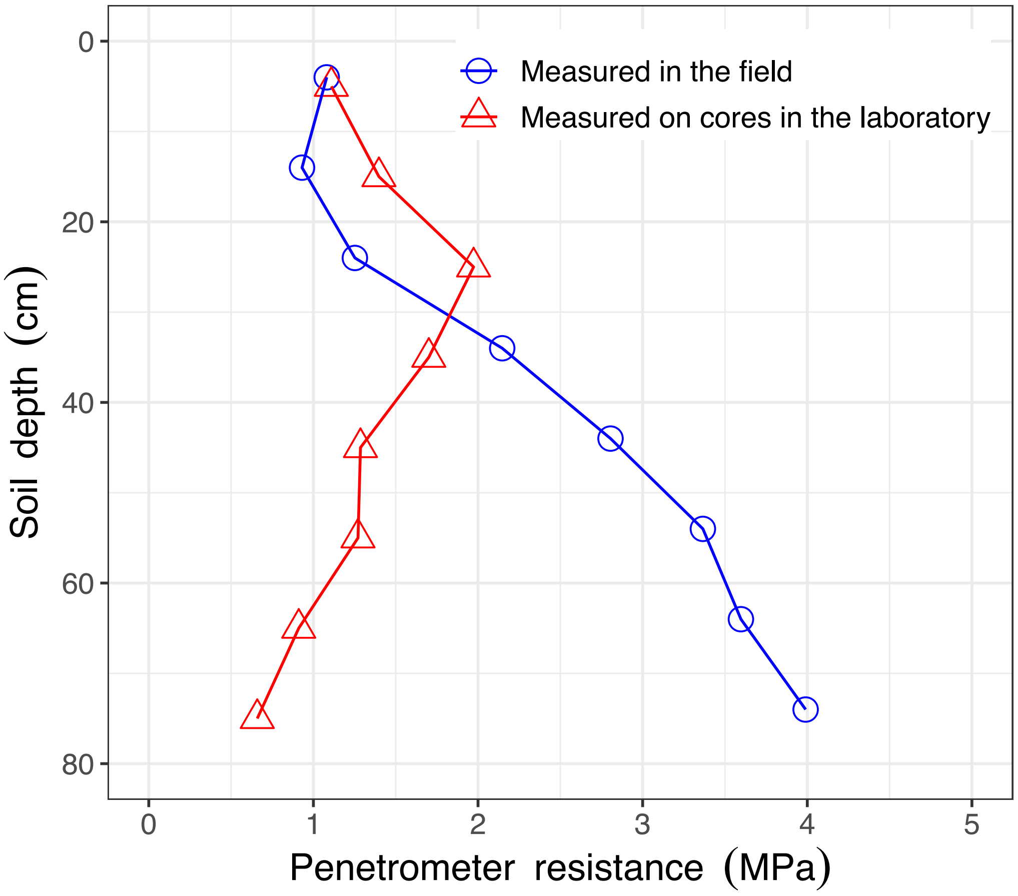

Figure S1. A comparison of penetrometer resistance measured in the field with penetrometer resistance measured in the laboratory on cores taken in the field

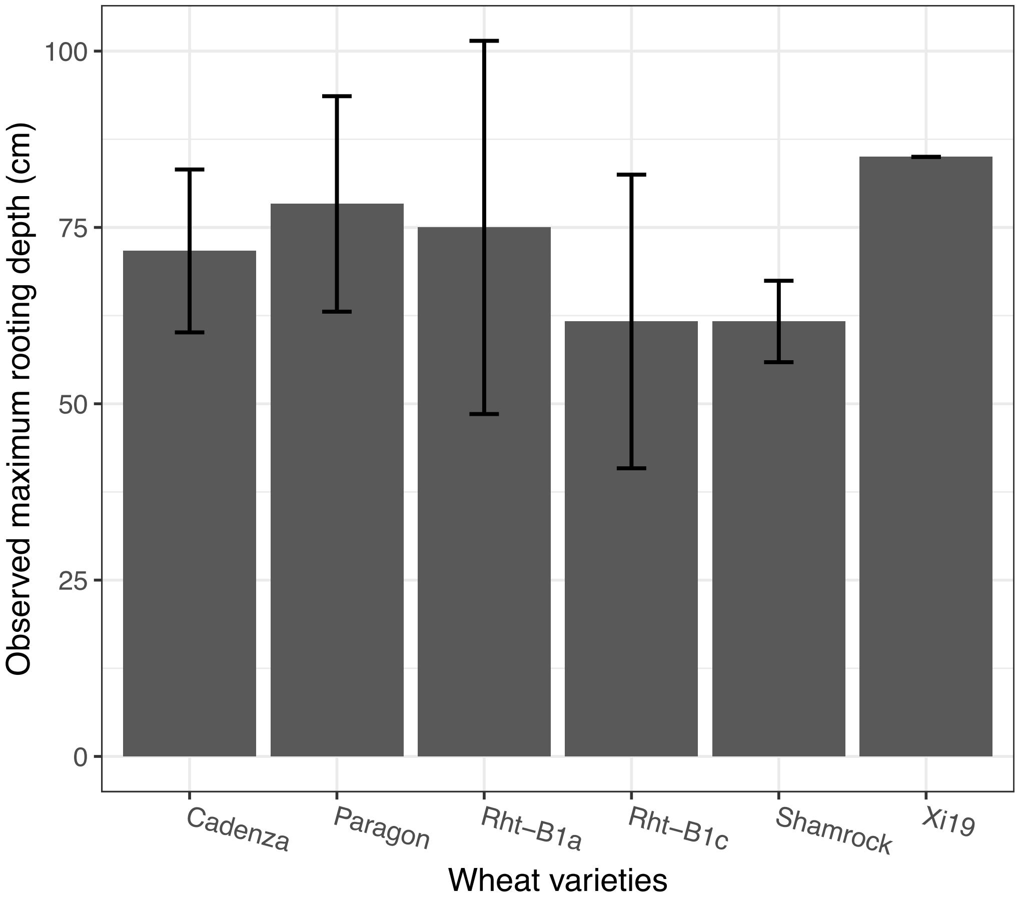

Figure S2. Observed maximum rooting depth of different wheat lines. Error bar indicate standard deviation.

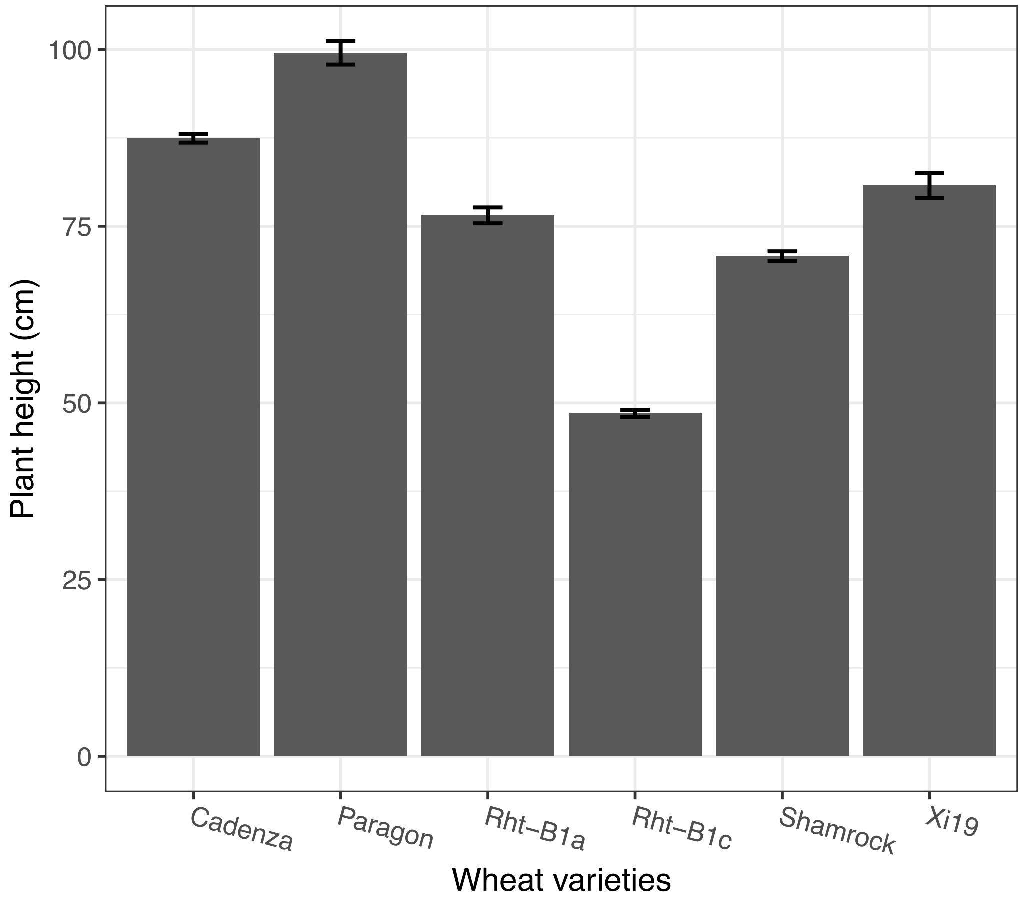

Figure S3. Plant height of different wheat lines. Error bar indicate standard deviation.
